# Cancer incidence in healthy Swedish peripheral blood stem cell donors

**DOI:** 10.1038/s41409-022-01617-6

**Published:** 2022-03-07

**Authors:** Simon Pahnke, Ulla Axdorph Nygell, Jan-Erik Johansson, Annika Kisch, Per Ljungman, Anna Sandstedt, Hans Hägglund, Gunnar Larfors

**Affiliations:** 1 Unit of Haematology, Department of Medical Sciences, Uppsala University, Uppsala, Sweden; 2 Unit for Apheresis, Clinical Immunology/Transfusion Medicine, Karolinska University Hospital, Stockholm, Sweden; 3 Department of Clinical Science, Intervention and Technology (CLINTEC), Karolinska Institutet, Stockholm, Sweden; 4 Department of Haematology and Coagulation, Sahlgrenska Academy, University of Gothenburg, Gothenburg, Sweden; 5 Department of Haematology, Skåne University Hospital; Institute of Health Sciences, Lund University, Lund, Sweden; 6 Department of Cellular Therapy and Allogeneic Stem Cell Transplantation, Karolinska Comprehensive Cancer Center, Karolinska University Hospital Huddinge, Stockholm, Sweden; 7 Division of Haematology, Department of Medicine, Huddinge, Karolinska Institutet, Stockholm, Sweden; 8 Department of Haematology, Linköping University Hospital, Linköping, Sweden

**Keywords:** Epidemiology, Cancer

## Abstract

Granulocyte colony-stimulating factor (G-CSF) has been used for over 20 years to obtain peripheral blood stem cells from healthy donors for allogeneic stem cell transplantation. Concerns have been raised about a potentially increased cancer incidence in donors after donation, especially regarding haematological malignancies. In a prospective Swedish national cohort study, we studied the cancer incidence after donation in 1082 Swedish peripheral blood stem cell donors, donating between 1998 and 2014. The primary objective was to evaluate if the cancer incidence increased for donors treated with G-CSF. With a median follow-up time of 9.8 years, the incidence of haematological malignancies was 0.85 cases per 1000 person-years, and did not significantly differ from the incidence in age-, sex- and residence-matched population controls (hazard ratio 1.70, 95% confidence interval (CI) 0.79–3.64, *p* value 0.17), bone marrow donors or non-donating siblings. The total cancer incidence for peripheral blood stem cell donors was 6.0 cases per 1000 person-years, equal to the incidence in matched population controls (hazard ratio 1.03, 95% CI 0.78–1.36, *p* value 0.85), bone marrow donors or non-donating siblings. In this study of healthy peripheral blood stem cell donors, the cancer incidence was not increased after treatment with G-CSF.

## Introduction

In the last 20 years, haematopoietic growth factors, such as granulocyte-colony stimulating factor (G-CSF), have become an integral part of the stem cell mobilisation procedure in most healthy blood stem cell donors.

G-CSF is an endogenous regulator of the proliferation and maturation of myeloid progenitor cells into mature neutrophils. The effect of G-CSF is mainly mediated through the transmembrane G-CSF receptor, found on both cells at early stages of haematopoiesis (hematopoietic stem cells and myeloid progenitors) and mature cells (neutrophilic granulocytes, monocytes, and lymphocytes). The receptor is also expressed in various non-hematopoietic cells including cardiovascular, neuronal, endothelial, and placental cells [1, 2]. The G-CSF receptor is expressed in malignant myeloid cells and has also been found to be expressed and possibly affect cell survival and proliferation in some solid tumours [3–5].

The short-term health risks associated with the use of G-CSF for stem cell mobilisation in healthy donors have been well described and are generally considered acceptable [6–9]. However, concerns have been raised regarding potentially increased risks of haematological malignancies and solid cancers [10, 11]. Several large studies of healthy donors have failed to show an increased short-term risk of developing haematological malignancies or any cancer after G-CSF treatment, but there is a lack of long-term safety data [8, 12, 13].

### Aim

Using a prospective Swedish national cohort study of peripheral blood stem cell (PBSC) donors, we aimed to investigate if the long-term risk of developing haematological malignancies or any cancer was increased after treatment with G-CSF.

We followed 1082 Swedish first-time PBSC donors, donating between 1998 and 2014, by combining data from several national Swedish population-based registers. The median follow-up time was 9.8 years. Their cancer incidence was compared to that of 5299 age-, sex- and residence-matched controls, 850 bone marrow donors, and 1115 full and half siblings.

## Methods

### Data collection

**Fig. 1:**
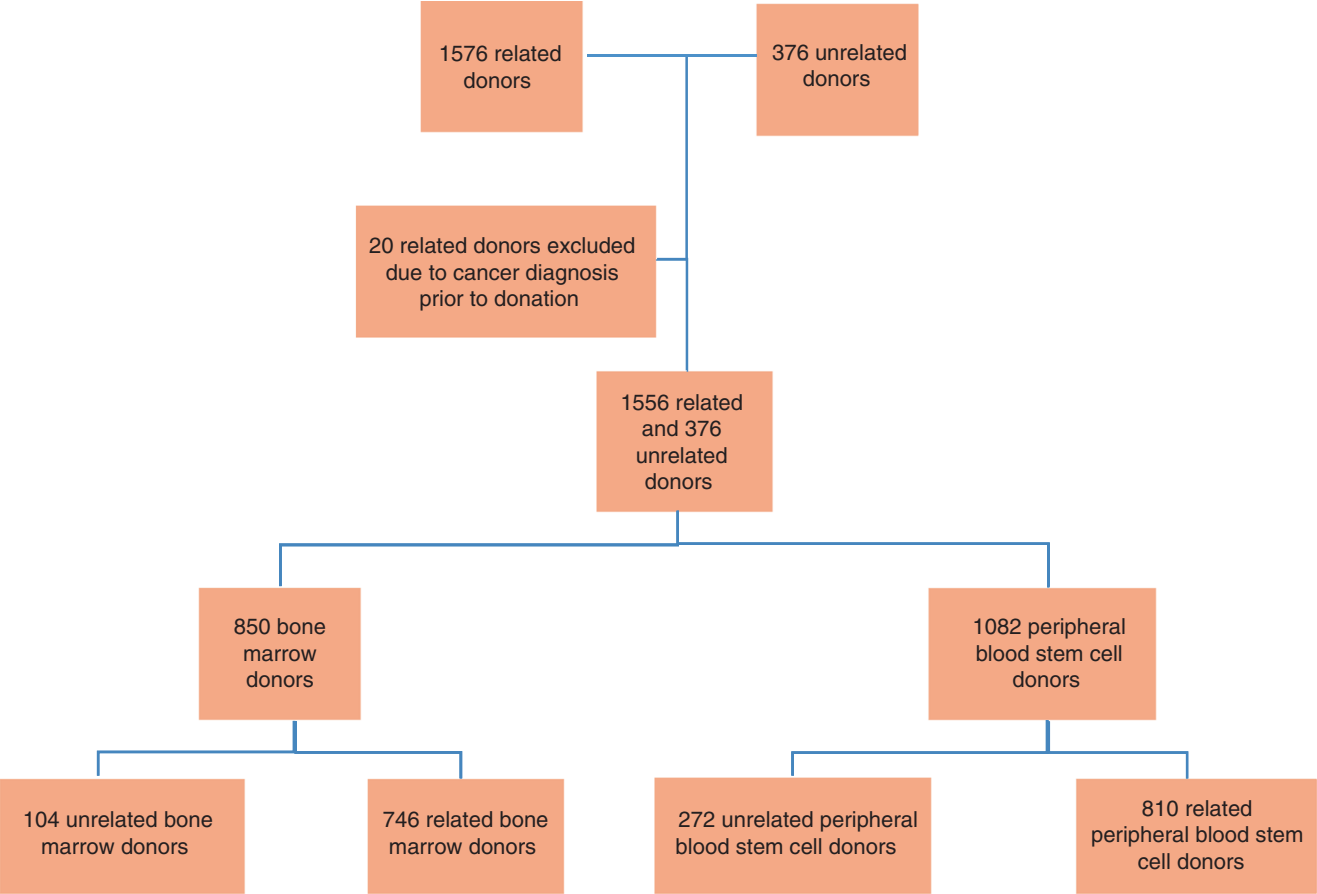
Inclusion of stem cell donors in the study. Distrubution of donors included in the study, according to relation to recipient and stem cell source.

A cohort of related (donating to a sibling, child or parent) haematopoietic stem cell donors donating in 1977–2014 was collected from all six Swedish centres for allogeneic stem cell transplantation (Fig. 1). BM donors donated between 1977 and 2014 and PBSC donors between 1998 and 2014.

A total of 1576 first-time related donors with complete records of personal national identification number, stem cell source and donation date were identified. For 19 additional donors, not all necessary information could be verified and they were excluded from the study. Donors (*n* = 20) and controls (*n* = 162) with any cancer diagnosis (except cancer in situ) before their donation date were excluded from the analysis of cancer incidence, which resulted in 1555 related donors included in the study.

An additional 376 unrelated Swedish donors, donating in 1998–2014 and previously included in the Nordic Register for Haematopoietic Stem cell Donors, were also included in the study [9].

A standard dosing of filgrastim G-CSF 1 ME/kg/day for 4–5 days prior to apheresis is generally used by Swedish donation centres [9].

A database was created by linking data from four Swedish national population-based registers, *the Swedish Cancer Register, the Swedish Multi-Generation Register, the Swedish Patient Register*, and *the Swedish Cause of Death Register*, containing data on all incident cancers for the 1082 PBSC donors, 1115 siblings, and 850 bone marrow donors.

For each PBSC donor, up to five population based controls, with the same year of birth, sex, and county (in Swedish: *län*) of residence at the end of the year of donation, were drawn at random by Statistics Sweden from the general Swedish population, using the Total Population Register, 5299 controls in total [14]. The linkage was performed at the Swedish National Board for Health and Welfare using national identification numbers, which were removed before delivery of the datasets for statistical analyses.

### The Swedish Cancer Register

The Swedish Cancer Register, started in 1958, requires reporting of all malignancies diagnosed by both clinicians and pathologists and generally has more than 96% coverage of all cancer diagnoses in Sweden [15, 16]. Except in cases of multiple skin and urinary tract tumours in the same topological area, each tumour is counted once. The Swedish Cancer Register has used the following International Classification of Disease codes: (ICD)-7 since 1958, ICD-9 since 1987, SNOMED (ICD-O/2) since 1993 and ICD-O/3 since 2005. Any first-time haematological malignancy, or any cancer, after donation and before December 31 2015, except non-melanoma skin cancer and premalignant lesions (cancer in situ), was counted as an event in the analyses.

### The Swedish Multi-Generation Register

The identity of all the donors’ siblings was obtained from the Multi-Generation Register, held by Statistics Sweden. The register is derived from Swedish population statistics and includes information about the identity of parents, siblings and children for Swedish residents born 1932 or later and resident in Sweden at some point in time from 1961 and onwards [17].

### The Swedish Patient Register

The Swedish Patient Register is a national register that includes diagnoses in an ICD format from admissions to hospital since 1965, and from specialised outpatient care since 2001 [18]. The Patient Register was used to ensure that none of the recipients of allogeneic stem cell transplantation was included as a control in the sibling comparison.

### The Swedish Cause of Death Register

Since 1952, dates and causes of death are recorded in the Cause of Death Register [19]. In addition to providing causes of death, the register can also serve as an alternative source of cancer diagnoses, and was used to search for cancer diagnoses in research subjects with no previous diagnosis registered in the Cancer Register. The Cancer Register and the Cause of Death Register were together estimated to cover more than 98% of all cases of acute leukaemia in the Swedish population [20].

### Statistical analysis

For data analysis, the statistical software SAS version 9.4 for Windows (SAS Institute, Cary, NC, USA) was used.

Cancer incidences for donors and their comparison groups were modelled through multivariable Cox regression [21], using the SAS procedure PHREG [22]. Sex (male/female), age at donation and relationship to recipient (related/unrelated) were included as potential confounding factors.

Relative risks, compared with controls, bone marrow donors and siblings, were estimated as hazard ratios with 95% CIs and results are presented both as crude event rates and in models adjusted for potential confounding factors.

### Power considerations

Prior to obtaining data for the study, we used data from Cancer Incidence in Sweden [23] to try to estimate the statistical power of the proposed study with regard to cancer. We estimated that we would have data on 1500 PBSC donors in Sweden, with 15,000 person-years of follow-up. We based our estimate on a yearly cancer incidence of 500 per 100,000 person-years of follow-up, which is the reported rate in Sweden at age 50 years. This would indicate that we could expect 75 new cancer cases in the cohort. With a power of 0.8 and an alpha level of 0.05, we would then have been able to detect relative risks of any cancer of 1.33 or higher in the study.

### Ethical review

The study was approved by the regional ethical review boards of Stockholm, 98–259, and Uppsala, 2016–497.

## Results

### Donor characteristics

The analyses encompassed 1932 first-time donors (1556 related and 376 unrelated), donating in 1977–2014. A total of 5 299 population-based controls for PBSC donors were identified, matched for sex, age, and highest education level reached, as seen in Table 1.

**Table 1:** Characteristics of peripheral blood stem cell (PBSC) donors, population controls, bone marrow donors, and siblings.

Characteristic	PBSC donors	Population controls^a^	Bone marrow donors	Siblings
	*N* (%)	*N* (%)	*N* (%)	
Number	1082	5 299	850	1 115
Sex				
Male	634 (59)	3 107 (59)	447 (53)	558 (52)
Female	448 (41)	2 192 (41)	403 (47)	537 (48)
Age at donation, years				
0–18	16 (1)	80 (2)	294 (35)	59 (5)
18–29	159 (15)	790 (15)	165 (19)	131 (12)
30–39	263 (24)	1301 (25)	184 (22)	140 (13)
40–49	294 (27)	1455 (27)	136 (16)	242 (22)
50–59	222 (21)	1078 (20)	59 (7)	319 (29)
60–69	114 (11)	531 (10)	12 (2)	192 (17)
70–79	14 (1)	64 (1)	-	32 (3)
Median	43.8	43.6	27.8	49.4
Min–Max	1.7–76.3	1.7–76.3	0.3–68.9	0–77.5
Relationship to recipient				
Related	810 (75)		746 (88)	1 115
Unrelated	272 (25)	5299	104 (12)	
Year of donation				
1970–1979			2 (<1)	
1980–1989			232 (27)	
1990–1999	115 (11)		327 (38)	142 (13)
2000–2009	689 (64)		198 (23)	703 (63)
2010–2014	278 (25)		91 (11)	270 (26)
Highest education level reached (by 2015)^b^	missing = 244	missing = 1180	missing = 195	missing = 274
Primary	52 (6)	290 (7)	38 (6)	50 (6)
Secondary	124 (15)	709 (17)	108 (16)	107 (19)
Post-secondary/non-tertiary	396 (48)	1907 (46)	326 (49)	438 (52)
Tertiary/Master/Doctoral	266 (32)	1213 (29)	183 (28)	246 (29)

^a^Matched for age, sex and county of residence of PBSC donors.

^b^Data available starting from 1990.

As a separate control population, with the benefit of having shared genetic backgrounds with the donors and likely similar socioeconomic backgrounds, all 1115 living and cancer-free (at time of donation) siblings of PBSC donors with at least one non-recipient sibling (*n* = 492) were identified from population registers.

Bone marrow donation was more common among related donors and younger donors. Especially paediatric donors were much more likely to donate bone marrow, with 95% of donors younger than 18 years donating bone marrow. Inversely, 91% of donors of age above 60 years, and all above 70 years, donated PBSC.

Before the 1990s, only bone marrow donation took place; a gradual shift towards an increasing proportion of PBSC donation occurred after the introduction of G-CSF. During the last years of the study (2010–2014), the proportion of PBSC donors was around 75%.

## Donor cancer incidence

### Comparison with population-based controls

**Fig. 2:**
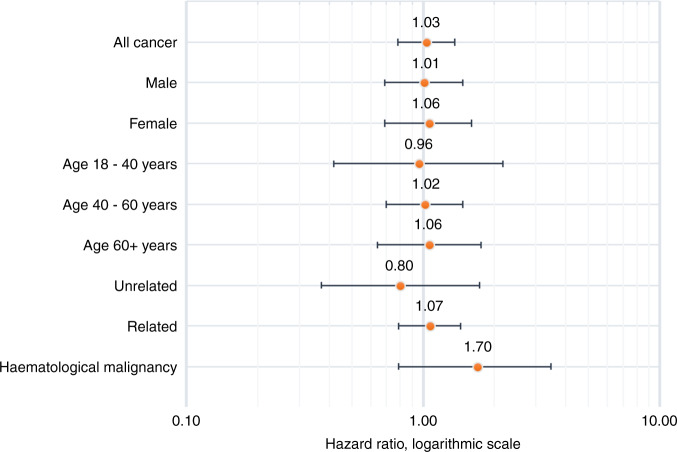
Cancer incidence rates compared with population controls. Cancer incidence rate hazard ratios and 95% confidence intervals for peripheral blood stem cell donors compared with age-, sex- and residency-matched population controls.

Among 1082 PBSC donors, with a median follow-up time of 9.8 years (min: 0.3, max: 21.3), a malignancy was diagnosed in 63 individuals (5.8%), as seen in Table 2. The event rate of 6 cancer cases per 1000 person years did not differ from that of age-, sex-, and residence-matched controls, where 282 (5.3%) cancer cases were detected among 5299 controls, 5.6 cases per 1000 person-years, hazard ratio 1.05 (95% CI 0.80–1.37, *p* value 0.75). Similarly, further subgroup analyses by age groups, sex or donor relationship to recipient did not show any difference in cancer incidence rates between donors and their matched controls (Fig. 2). The distribution of cancer according to location is provided as supplementary information, Appendix 1.

**Table 2:** Cancer events in peripheral blood stem cell donors compared with age-, sex-, and residence-matched controls.

	PBSC donors			Matched population controls					
Variable	*N* (%)	Number of events	Events/1000 person-years	*N* (%)	Number of events (%)	Events/1000 person-years	Hazard ratio	95% confidence interval	*P* value
Any cancer	1 082	59 (5.45)	5.65	5 299	285 (5.38)	5.58	1.03	0.78–1.36	0.85
Sex									
Male	634 (59)	33 (5.2)	5.37	3 107	156 (5.0)	5.24	1.01	0.69–1.47	0.98
Female	448 (41)	26 (5.8)	6.05	2 192	129 (5.9)	6.2	1.05	0.69–1.60	0.81
Age at donation, years									
0–18	16 (1)	0	0	80	1 (1.3)	0.95			
18–40	422 (38)	7 (1.7)	1.41	2 091	33 (1.6)	1.56	0.96	0.42–2.17	0.92
40–60	516 (49)	34 (6.6)	7.31	2 533	166 (6.6)	7	1.02	0.70–1.47	0.93
60+	128 (12)	18 (14.1)	20.17	594	85 (14.3)	18.42	1.06	0.64–1.76	0.83
Relationship to recipient									
Unrelated	272 (25)	8 (2.9)	3.03	1 348	31 (2.3)	3.97	0.8	0.37–1.73	0.57
Related	810 (75)	51 (6.3)	7.04	3 951	254 (6.4)	6.77	1.07	0.79–1.44	0.68
Haematological malignancy	1082	9 (0.8)	0.85	5 299	25 (0.5)	0.48	1.7	0.79–3.64	0.17

Nine PBSC donors (0.83%) were diagnosed with a haematological malignancy during the follow-up period. Diagnoses included one case each of acute myeloid leukaemia, Hodgkin’s lymphoma, diffuse large B-cell lymphoma, essential thrombocythemia, myelofibrosis, follicular lymphoma, and enteropathy-associated T-cell lymphoma, as well as two cases of chronic lymphocytic leukaemia.

The resulting haematological malignancy incidence rate of 0.86 cases per 1000 person-years of follow-up was not significantly different from that in the control group (26 cases (0.51%), incidence rate 0.51 cases per 1000 person-years, hazard ratio 1.62 (95% CI 0.76–3.46, *p* value 0.21).

### Comparison with bone marrow donors

**Fig. 3:**
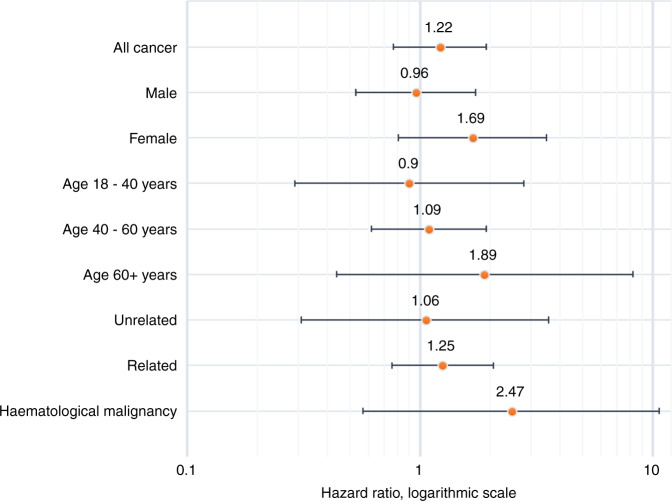
Cancer incidence rates compared with bone marrow donors. Age- and sex-adjusted cancer incidence rate hazard ratios and 95% confidence intervals for peripheral blood stem cell donors compared with bone marrow donors.

The cancer incidence in the 1082 PBSC donors was also compared with that of 850 bone marrow donors, with a median follow-up time of 20 years (0.2–36.1 years), Table 3. Fifty-nine bone marrow donors were diagnosed with a malignancy (6.96%). The hazard ratio of being diagnosed with a malignancy was 1.22 (95% CI 0.77–1.93, *p* value 0.40) for PBSC donors compared with bone marrow donors, when adjusted for age and sex, Fig. 3. Analyses by age groups, sex and relationship to donor revealed no subgroup with a statistically significant difference in cancer incidence rate. Inclusion of sociodemographic markers, such as highest educational attainment and residence, did not affect the results of the analysis (results not shown).

**Table 3:** Cancer events in peripheral blood stem cell donors (PBSC) compared with bone marrow donors.

	PBSC donors			Bone marrow donors					
Variable	*N* (%)	Number of events (%)	Events/1000 person-years	*N*	Number of events (%)	Events/1000 person-years	Hazard ratio	95% confidence interval	*P* value
Any cancer	1 082	59 (5.45)	5.65	850	60 (7.1)	3.72	1.22	0.76–1.96	0.40
Sex									
Male	634 (59)	33 (5.2)	5.37	447	31 (6.9)	3.67	0.91	0.50–1.65	0.75
Female	448 (41)	26 (5.8)	6.05	403	29 (7.2)	3.78	1.88	0.88–4.04	0.11
Age at donation, years									
0–18	16 (1)	0	0	294	5 (1.7)	0.88	-		
18–40	422 (38)	7 (1.7)	1.41	349	21 (6.0)	3.11	1.01	0.34–2.98	0.99
40–60	516 (49)	34 (6.6)	7.31	195	32 (16.4)	9.03	1.10	0.61–1.97	0.76
60+	128 (12)	18 (14.1)	20.17	12	2 (16.7)	11.66	1.50	0.34–6.60	0.60
Relation to recipient									
Unrelated	272 (25)	8 (2.9)	3.03	104	3 (2.9)	2.37	1.41	0.37–5.36	0.62
Related	810 (75)	51 (6.3)	7.04	746	57 (7.6)	3.84	1.19	0.72–1.98	0.50
Haematological malignancy	1 082	9 (0.8)	0.86	850	4 (0.5)	0.25	2.48	0.57–10.75	0.22

Only four bone marrow donors (0.47%) were diagnosed with a haematological malignancy during the long follow-up period. The hazard ratio for PBSC donors of being diagnosed with a haematological malignancy compared with bone marrow donors was 2.47 (95% CI 0.57–10.66, *p* value 0.11), adjusted for age and sex.

### Comparison with non-donating siblings

A separate comparison was made between sibling donors and their non-donating siblings. A total of 810 sibling PBSC donors were identified. Only donors with a non-transplant-recipient sibling alive at the time of the donation, where neither the donor nor the sibling had any cancer prior to donation, were included in the analysis, resulting in a group of 492 sibling donors and 1115 siblings, Table 4. Thirty-three donors (6.71%) were diagnosed with any cancer, seven (1.02%) of which were haematological malignancies, compared with 82 cancer cases among siblings (7.35%), with eight (0.72%) haematological malignancies.

**Table 4:** Cancer events in related peripheral blood stem cell (PBSC) donors compared with their siblings.

	PBSC donors			Siblings					
Variable	*N*	Number of events (%)	Events/1000 person-years	*N*	Number of events (%)	Events/1000 person-years	Hazard ratio	95% confidence interval	*P* value
Any cancer	492	33 (6.7)	7.48	1 115	82 (7.3)	7.61	1.06	0.75–1.51	0.74
Sex									
Male	282	17 (6.0)	7.46	578	39(6.8)	7.01	1.13	0.69–1.86	0.62
Female	210	16 (7.6)	7.52	537	43 (8.2)	8.01	0.97	0.58–1.62	0.91
Age at donation, years									
0–18	14	0	-	59	0	-	-		
18–40	131	2 (1.5)	0.98	271	6 (2.2)	2.06	0.49	0.098–2.42	0.38
40–60	270	21 (7.8)	8.45	561	45 (8.0)	8.38	1.04	0.65–1.65	0.88
60+	80	10 (12.5)	20	224	31 (13.8)	17.37	1.21	0.67–2.17	0.53
Haematological malignancy	492	6 (1.2)	1.05	1 115	8 (0.7)	0.74	1.48	0.53–4.11	0.45

**Fig. 4:**
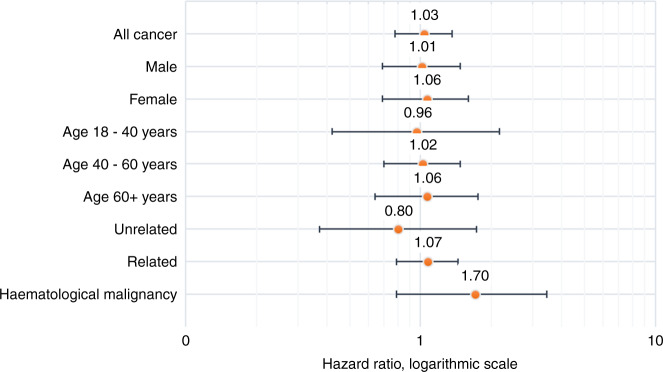
Cancer incidence rates compared with non-donating siblings. Age- and sex-adjusted cancer incidence rate hazard ratios and 95% confidence intervals for related peripheral blood stem cell donors compared with their non-donating siblings.

The hazard ratio for PBSC donors to be diagnosed with any cancer during follow-up was 1.06 (95% CI 0.75–1.51. *p* value 0.74) and for haematological cancer 1.48 (95% CI 0.53–4.11, *p* value 0.45), compared with their non-donating siblings, Fig. 4.

## Discussion

The concern regarding potential effects of G-CSF on cancer development in healthy donors rests mainly on theoretical reasoning regarding the impact of stimulating haematopoietic stem/progenitor cells. Studies of epigenetic and genetic effects of G-CSF treatment and studies of long-term effects of G-CSF treatment for nonmalignant diseases have reported conflicting findings [24–30].

### Previous studies of malignancies in healthy donors

Several studies have found sporadic cases of haematological malignancies after PBSC donation. Makita et al. first reported a case of acute myeloid leukaemia (AML) in a related PBSC donor and Bennet et al. subsequently identified two additional cases [11, 31]. Other case reports describe singular cases of association of G-CSF use with myeloma infiltration of muscle, leukaemic nodules or pronounced lymphadenopathy at G-CSF injection site, marked progression of myeloma and transformation of aleukaemic leukaemia to leukaemia [32–36]. Furthermore, a retrospective single-centre review of 8005 German PBSC donors by Hölig et al. identified two AML cases, resulting in a significantly increased standardised incidence ratio of 11.4 (95% CI 1.4–41.2), and three cases of Hodgkin’s lymphoma, standardised incidence ratio 6.2 (95% CI 1.3–18.0) [37]. However, the comparison in that study was made to reported incidence ratios for the total German population and the authors issued a caution regarding interpretation of their findings.

Numerous other studies have not shown an increase in the incidence of either haematological malignancies or cancer in general [6, 7, 12, 13, 38–49]. A 2011 meta-analysis of eight of these studies, including 40,717 donors followed for 151,016 person-years, identified three AML cases. The resulting incidence rate of 2 per 100,000 years was below the incidence rate of 3.5 per 100,000 person-years reported for the general population in the United States [50].

### Limitations of previous studies

The results from previous studies are reassuring, but there are some aspects of the study designs used that makes it difficult to draw definitive conclusions regarding the potential risk for malignancies associated with G-CSF treatment.

First, most studies have followed donors for a median of less than four years. Studies of secondary malignancies after other treatments, i.e., chemotherapy and/or radiation, have shown increased secondary malignancy incidence rates for up to 30 years after treatment, and median time to diagnosis is usually in the range 5–15 years, generally somewhat shorter for haematological malignancies [51–54].

Second, by relying primarily on surveys of either the transplant centres or the donors themselves for reporting diagnosed malignancies, most studies are limited by loss to follow-up over time, sometimes reaching more than 60% at 5 years. The possibility that donors who develop malignancies might be even more likely to be lost to follow-up or survey completion adds further uncertainty regarding the reliability of the findings.

### Our findings

In our prospective, register-based cohort study, we present long-term follow-up data on the cancer incidence in healthy donors after PBSC donation. With a median follow-up time of 9.8 years, and a total of 10,435 person-years, the total risk of cancer for PBSC donors was not different to that of an age-, sex-, and residence-matched population, Swedish bone marrow donors, or the donors’ siblings. The incidence rate of haematological cancers for PBSC donors was reassuringly low (0.86/1000 person-years) even after long-term follow-up, and not significantly higher than that of matched controls, bone marrow donors and the donors’ siblings.

The prospective design, the fairly large study population, the population-based inclusion of practically all related donors since the start of stem cell transplantation in Sweden in 1975, coupled with a long follow-up time and the low loss-to-follow-up that comes with using Swedish national registers for cancer incidence, are important factors improving the reliability of the results.

However, there are also significant limitations to our study. Because the population size of Sweden is less than 10 million inhabitants, the study is not powered to adequately evaluate incidence rate changes for individual haematological malignancies, or even to completely rule out a small, but perhaps clinically relevant, incidence rate increase for all haematological cancers.

Attempts have been made to calculate the study size needed to assess the possibility of an increased risk of haematological malignancies in healthy donors. Hasenclever calculated that 2000 donors needed to be followed for 10 years to detect even a tenfold increase in acute myeloid leukaemia [55], about twice as many as in this study.

Based on the CIs obtained in the study, the total incidence rate of any cancer for PBSC donors treated with G-CSF can with high certainty be said to be below 1.38 times that of well-matched general population controls and, for haematological cancer, below 3.61 times.

## Conclusion

In a prospective national cohort study of 1082 Swedish PBSC donors, we found no significant difference in overall or haematological cancer incidence, compared with in matched controls, bone marrow donors or the donors’ siblings, even with a long median follow-up time of 9.8 years. These results add new information to the existing knowledge about potential health risks for donors and should be of importance when counselling prospective donors.

## Supplementary information

Appendix 1
