# Supplementary material for: Cancer incidence in healthy Swedish peripheral blood stem cell donors
Source: Bone Marrow Transplant. 2022 Mar 7;57(5):795–802. doi: 10.1038/s41409-022-01617-6 (PMC9090628; doi:10.1038/s41409-022-01617-6)
Supplement: Supplementary file 1 — Appendix 1 [file 41409_2022_1617_MOESM1_ESM.pdf]

	Cancer frequency in PBSC donors (n=1082)		Cancer frequency in matched population controls (n=5299)	
**Cancer type**	**Frequency**	**Percent**	**Frequency**	**Percent**
Head/neck cancer	3	0.28	9	0.17
Gastrointestinal cancer	13	1.2	51	0.96
Lung cancer	2	0.18	18	0.34
Breast cancer	8	0.74	47	0.89
Gynaecological cancer	3	0.28	19	0.36
Prostate cancer	11	1.02	56	1.06
Urological cancer, incl. male genital cancer	3	0.28	18	0.34
Melanoma skin cancer	3	0.28	17	0.32
Cancers of the nervous system	3	0.28	10	0.19
Endocrine cancer	1	0.09	10	0.19
Haematological malignancies	9	0.83	26	0.49
Other cancers*	-	-	4	0.08
No cancer	1023	94.54	5014	94.62

Appendix 1:
Cancer frequencies of major cancer types for PBSC donors and their matched population controls.
*Including two cases of sarcoma and one case each of adenocarcinoma and undifferentiated carcinoma with missing primary tumor localisation.
